# *RNF43* and *ZNRF3* are commonly altered in serrated pathway colorectal tumorigenesis

**DOI:** 10.18632/oncotarget.12130

**Published:** 2016-09-20

**Authors:** Catherine E. Bond, Diane M. McKeone, Murugan Kalimutho, Mark L. Bettington, Sally-Ann Pearson, Troy D. Dumenil, Leesa F. Wockner, Matthew Burge, Barbara A. Leggett, Vicki L.J. Whitehall

**Affiliations:** ^1^ Conjoint Gastroenterology Laboratory, QIMR Berghofer Medical Research Institute, Brisbane, Queensland, Australia; ^2^ Signal Transduction Laboratory, QIMR Berghofer Medical Research Institute, Brisbane, Queensland, Australia; ^3^ Envoi Specialist Pathologists, Brisbane, Queensland, Australia; ^4^ School of Medicine, University of Queensland, Brisbane, Queensland, Australia; ^5^ Cancer and Population Studies, QIMR Berghofer Medical Research Institute, Brisbane, Queensland, Australia; ^6^ Department of Oncology, Royal Brisbane and Women's Hospital, Brisbane, Queensland, Australia; ^7^ Department of Gastroenterology and Hepatology, Royal Brisbane and Women's Hospital, Brisbane, Queensland, Australia; ^8^ Pathology Queensland, Brisbane, Queensland, Australia

**Keywords:** RNF43, colorectal cancer, BRAF, MSI, Wnt signalling

## Abstract

Serrated pathway colorectal cancers (CRCs) are characterised by a *BRAF* mutation and half display microsatellite instability (MSI). The Wnt pathway is commonly upregulated in conventional CRC through *APC* mutation. By contrast, serrated cancers do not mutate *APC*. We investigated mutation of the ubiquitin ligases *RNF43* and *ZNRF3* as alternate mechanism of altering the Wnt signal in serrated colorectal neoplasia. *RNF43* was mutated in 47/54(87%) *BRAF* mutant/MSI and 8/33(24%) *BRAF* mutant/microsatellite stable cancers compared to only 3/79(4%) *BRAF* wildtype cancers (p<0.0001). *ZNRF3* was mutated in 16/54(30%) *BRAF* mutant/MSI and 5/33(15%) *BRAF* mutant/microsatellite stable compared to 0/27 *BRAF* wild type cancers (p=0.004). An *RNF43* frameshift mutation (X659fs) occurred in 80% *BRAF* mutant/MSI cancers. This high rate was verified in a second series of 25/35(71%) *BRAF* mutant/MSI cancers. *RNF43* and *ZNRF3* had lower transcript expression in *BRAF* mutant compared to *BRAF* wildtype cancers and less cytoplasmic protein expression in *BRAF* mutant/MSI compared to other subtypes. Treatment with a porcupine inhibitor reduced *RNF43*/*ZNRF3* mutant colony growth by 50% and synergised with a MEK inhibitor to dramatically reduce growth. This study suggests inactivation of *RNF43* and *ZNRF3* is important in serrated tumorigenesis and has identified a potential therapeutic strategy for this cancer subtype.

## INTRODUCTION

The majority of colorectal cancers up-regulate the Wnt signalling pathway [1]. This is commonly due to mutation of the *APC* gene which is uncommonly mutated in *BRAF* mutant serrated pathway cancers [2]. Serrated neoplasia accounts for 25-30% of all colorectal cancers and these arise from sessile serrated adenomas (SSAs) or traditional serrated adenomas (TSAs) [3, 4]. Approximately half of all *BRAF* mutant serrated pathway cancers will methylate the mismatch repair gene *MLH1* and develop microsatellite instability (MSI) [5, 6], whilst the remainder are microsatellite stable (MSS). A recent study reported germline mutation of the upstream Wnt inhibitor *RNF43* in a minority of subjects with serrated polyposis [7, 8], providing a potential alternative to *APC* mutation as a mechanism for altering the Wnt signal in serrated neoplasia.

*RNF43* is a transmembrane E3 ubiquitin ligase that is up-regulated in response to accumulation of β-catenin following increased Wnt signalling [8, 9]. Together with its functional homologue, *ZNRF3*, it localizes to the plasma membrane and targets *Frizzled* (*FZD*), a key receptor of the Wnt ligand, for ubiquitination and lysosomal degradation [8]. *RNF43* and *ZNRF3* are highly expressed in murine intestinal stem cells, and deletion of these renders cells hypersensitive to Wnt ligand secretion, resulting in intestinal adenomas with strong expression of β-catenin [8].

Previous studies have found *RNF43* to be functionally mutated in pancreatic cysts [10, 11] and mucinous ovarian cancer [12]. Frequent frameshift mutations in *RNF43* have been associated with MSI gastric, colorectal and endometrial cancers [13–15]. Loss of heterozygosity at the *RNF43* locus, 17q22, has been observed in the pancreatic cyst, intraductal papillary mucinous neoplasms (IPMNs) [10, 16] and copy number variations of *ZNRF3*, predominantly consisting of homozygous deletions at its 22q12.1 locus, have been found in adrenocortical carcinoma and osteoblastoma [17–19].

Release of the Wnt ligand from a signalling cell is dependent on the molecule Porcupine (*PORCN*). *PORCN* facilitates post-translational palmitoylation of the Wnt ligand which is necessary for its transport out of the cell and subsequent recognition by a *FZD* receptor on a receiving cell. Treatment with a Porcupine inhibitor reduces growth of *RNF43* mutant pancreatic cancer cell lines, where proliferation is dependent on endogenous Wnt signalling [20]. The porcupine inhibitor LGK974 has been shown to dramatically reduce growth of pancreatic cancer cells that carry an *RNF43* mutation [20].

We analysed *RNF43* and *ZNRF3* for mutation, copy number variation and expression in a large number of colorectal cancers stratified for molecular subtype. We hypothesized that in serrated pathway cancers where *APC* mutation is uncommon, inactivation of *RNF43* and/or *ZNRF3* would present an alternate mechanism for activating the Wnt signal.

## RESULTS

### Clinicopathological data of cancer cohorts

Cancers displayed typical clinicopathological features when stratified for molecular subtype as previously described [21–23]. Patients with *BRAF* mutant/MSI cancers presented at significantly older ages compared to both MSS subtypes. *BRAF* mutant cancers were predominantly present in the proximal colons of females and displayed the methylator phenotype more commonly than *BRAF* wild type cancers (Table 1).

**Table 1:** Clinical and Molecular data of Cancers

	BRAF mutant/MSI	BRAF mutant/MSS	BRAF wild type /MSS	p value	P Value^A^	P Value^B^	P Value^C^
*RBWH cancers:*							
n	54	33	79	-	-	-	-
Average age (yrs)	74.6	68.4	68.2	0.005	0.026	0.003	0.900
Female gender	42/54 (77.8%)	17/33 (51.5%)	33/79 (18.4%)	0.0002	0.017	<0.0001	0.407
Proximal site	41/43 (95.3%)	17/24 (70.8%)	15/63 (23.8%)	<0.0001	0.008	<0.0001	0.0001
CIMP high	54/54 (100%)	32/33 (96.7%)	12/79 (15.1%)	<0.0001	0.379	<0.0001	<0.0001
*Envoi cancers:*							
n	63	37	44	-	-	-	-
Average age (yrs)	77.9	72.6	63.6	<0.0001	0.01	<0.0001	0.004
Female gender	37/57 (64.9%)	26/37 (70.2%)	13/28 (46.4%)	0.12	0.66	0.16	0.07
Proximal site	53/57 (93.0%)	30/37 (81.1%)	9/28 (32.1%)	<0.0001	0.10	<0.0001	<0.0001
CIMP high	59/63 (93.7%)	23/35 (65.7%)	0/35 (0%)	<0.0001	0.001	<0.0001	<0.0001

A P Value: P value between *BRAF* mutant/MSI and *BRAF* mutant/MSS cohorts;

B P Value: P value between *BRAF* mutant/MSI and *BRAF* wild type cohorts;

C P Value: P value between *BRAF* mutant/MSS and *BRAF* wild type cohorts

### Mutation frequencies of *RNF43* and *ZNRF3* vary by molecular subtype

The presence of *RNF43* mutations was examined in 54 *BRAF* mutant/MSI, 33 *BRAF* mutant/MSS and 79 *BRAF* wild type cancers. *RNF43* was frequently mutated in *BRAF* mutant/MSI cancers (47/54; 87.0%). *BRAF* mutant/MSS cancers had a moderate proportion of *RNF43* mutations (8/33; 24.2%), whilst *BRAF* wild type cancers had the least frequent mutations compared to the *BRAF* mutant subgroups (3/79, 3.8%) (p<0.0001) (Table 2).

**Table 2:** Frequency and type of *RNF43* mutations

Cohort	n	Number of cancers mutated	Total number of mutations	X659 frameshift mutations	X117 frameshift mutations	Other frameshift mutations	Mis/nonsense mutations
*BRAF* mutant /MSI	54	47 (87.0%)	65	43 (79.6%)	6 (11.1%)	9 (16.7%)	7 (13.0%)
*BRAF* mutant /MSS	33	8 (24.2%)	8	1 (3.0%)	0	5 (15.2%)	2 (6.1%)
*BRAF* wild type /MSS	79	3 (3.8%)	3	0	0	0	3 (3.8%)

P value for number of mutations: Overall p<0.0001; *BRAF* mutant/MSI vs *BRAF* mutant/MSS p<0.0001; *BRAF* mutant/MSI vs *BRAF* wild type p<0.0001; *BRAF* mutant/MSS vs *BRAF* wild type p=0.002

The most common type of *RNF43* mutation was a frameshift at nucleotide 659 (G7 repeat tract) in exon 9 that occurred in 43/54 (79.6%) *BRAF* mutant/MSI, in 1/33 (3.0%) *BRAF* mutant/MSS and 0/79 *BRAF* wild type cancers (Table 2). The high frequency of this particular mutation was further validated in the second series of formalin fixed cancers where it was found in 25/35 (71.4%) *BRAF* mutant/MSI, 1/12 (8.3%) *BRAF* mutant/MSS and 0/22 *BRAF* wild type cancers (p<0.0001).

**Figure 1:**
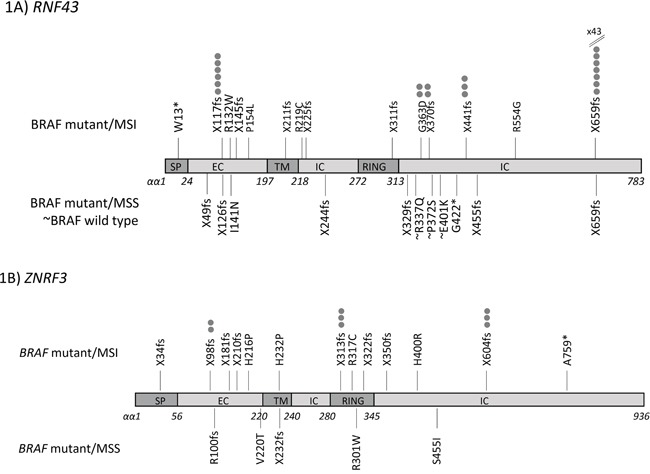
Mutation location map across coding sequences for A. *RNF43* and B. *ZNRF3* in colorectal cancer subtypes

The prevalence of other mutations is shown in Table 2 and Figure 1A. MSI cancers tended to show mutations in repeat tracts. In addition to the G659 (G7 deletion), recurrent frameshift mutations were present at R117 (C6 repeat tract) in exon 3 and affected 6/54 (11.1%) of *BRAF* mutant/MSI cancers. Other recurrent mutations in *BRAF* mutant/MSI cancers were X441fs (n=3), X370fs (n=2) and G363D (n=2). Eight *BRAF* mutant/MSS cancers (8/33, 24.2%) had *RNF43* mutations including 2 point mutations and 6 frameshift mutations that were located throughout the gene. *BRAF* wild type cancers had a minimal mutation rate of 3/79 (3.8%) and all were missense mutations in exon 9 (Table 2, Figure 1A).

*ZNRF3* mutations also associated with the *BRAF* V600E mutation (MSI: 16/54, 29.6%; MSS: 5/33, 15.2%, p=0.2) (Table 3, Figure 1B). There were no *ZNRF3* mutations in the *BRAF* wild type cohort (vs *BRAF* mutant/MSI p=0.0008; vs *BRAF* mutant/MSS p=0.06) (Table 3). Common *ZNRF3* frameshift mutations were seen at P313 (C5 deletion) and G604 (G6 deletion) with 3 of each seen in *BRAF* mutant/MSI cancers (Table 3). Point mutations were found in 5 *BRAF* mutant/MSI and 4 *BRAF* mutant/MSS cancers, and these were located along the length of the gene (Figure 1B). *RNF43* or *ZNRF3* mutations were not associated with other clinical or molecular parameters (Supplementary Table S1).

**Table 3:** Frequency and type of *ZNRF3* mutations

Cohort	n	Number of cancers mutated	Total number of mutations	Frameshift mutations	Mis/nonsense (%A) (%B)
*BRAF* mutant /MSI	54	16 (29.6%)	18	13 (24.1%)	5 (9.3%)
*BRAF* mutant /MSS	33	5 (15.2%)	5	1 (3.0%)	4 (9.1%)
*BRAF* wild type /MSS	27	0	0	0	0

P value for number of mutations: Overall p=0.004; *BRAF* mutant/MSI vs *BRAF* mutant/MSS p=0.2; *BRAF* mutant/MSI vs *BRAF* wild type p=0.008; *BRAF* mutant/MSS vs *BRAF* wild type p=0.06

Ten colorectal cancer cell lines were analysed for presence of an *RNF43* and *ZNRF3* mutation. *RNF43* mutations were found in five cell lines (RKO, SW48, DLD1, HCT116, LS174T) which are all MSI. RKO, SW48 and DLD1 harboured the X659fs mutation, with RKO showing a X659fs homozygous mutation. Additional mutations were seen in SW48 (X299fs) and DLD1 (L214M). HCT116 had a homozygous X117fs and a heterozygous X606fs mutation. LS174T had two missense mutations (K108E, R389H). RKO and LISP1 were the only cell lines harbouring *ZNRF3* mutations. RKO had 1 frameshift and 1 missense mutation (X249fs, G481W), whilst LISP1 had a missense mutation (H461R).

### *RNF43* and *ZNRF3* transcript expression is upregulated in *BRAF* wild type compared to *BRAF* mutant colorectal cancers

**Figure 2:**
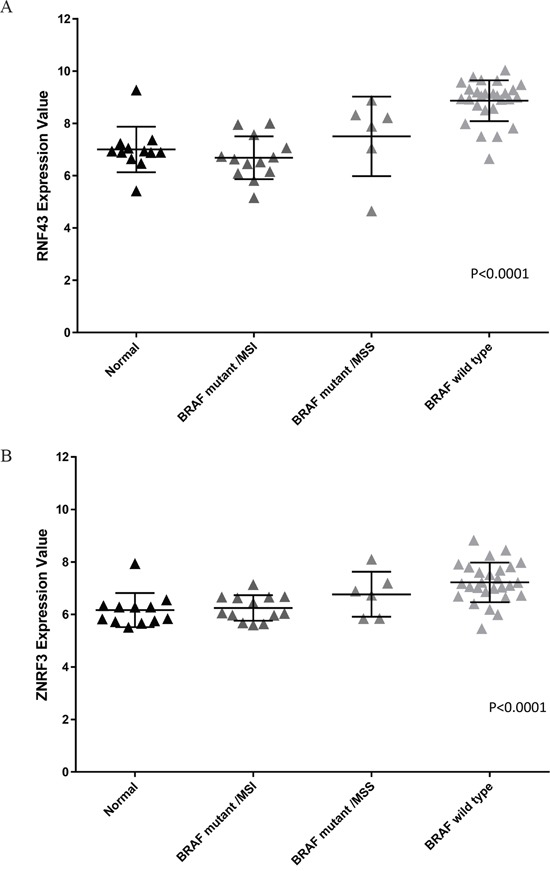
Transcript expression for A. *RNF43* and B. *ZNRF3* between cancer cohorts *BRAF* wild type cancers have increased expression of both *RNF43* and *ZNRF3* compared to *BRAF* mutant/MSI and *BRAF* mutant/MSS subgroups and normals (p<0.0001 for *RNF43* and *ZNRF3*).

From those cancers that were assessed for presence of mutation, a subset of cancers were examined for *RNF43* and *ZNRF3* transcript expression levels. Analysis revealed significantly higher expression for both *RNF43* and *ZNRF3* in *BRAF* wild type compared to *BRAF* mutant cancers and normal colorectal mucosa samples (p<0.0001 for both genes) (Figure 2A and 2B). *RNF43* transcript expression was lower in *RNF43* mutant compared to *RNF43* wild type cancers (mean of 6.91 and 8.49 respectively; p=0.0002) (Supplementary Table S2A). Cancers with an X659fs mutation had lower expression compared to cancers harbouring other mutations (average of 6.5 and 8.15 respectively; p=0.01) and compared to *RNF43* wild type cancers (p<0.0001) (Supplementary Table S2A). Similarly, transcript expression of *ZNRF3* mutant cancers was significantly lower than *ZNRF3* wild type cancers (average of 6.16 and 7.02 respectively; p=0.009) (Supplementary Table S2B).

### Structural variations in *RNF43* and *ZNRF3* between cohorts

At the 17q22 *RNF43* locus, *BRAF* mutant/MSS cancers had significantly more frequent deletion events (at 18/33, 54.5%) than *BRAF* wild type cancers (4/18, 22.2%; p=0.04). *BRAF* mutant/MSI cancers had a low rate of deletion (4/30, 13.3%), which was expected since MSI cancers are known to be diploid [22].

At the *ZNRF3* locus, 22q12.1, there was a high frequency of deletion events in MSS cancers (*BRAF* mutant/MSS: 16/33, 48.5%; *BRAF* wild type: 10/18, 55.6%). In addition to frequent loss of heterozygosity of *ZNRF3* occurring in the *BRAF* mutant/MSS cohort, one of these cancers harboured a homozygous deletion spanning approximately 170kb and including the C terminal half of the ZNRF3 locus. Again *BRAF* mutant/MSI cancers rarely showed deletion events at this locus (1/30, 3.3%).

### RNF43 cytoplasmic expression is reduced in *BRAF* mutant/MSI cancers

**Figure 3:**
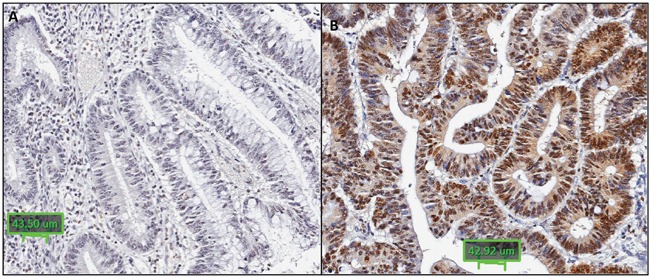
Representative images of RNF43 immunohistochemistry RNF43 immunohistochemistry showing: A. negative cytoplasmic and nuclear staining in a *BRAF* mutant/MSI cancer; B. positive cytoplasmic and nuclear staining in a *BRAF* wild type cancer (x20).

Immunohistochemical staining was performed to detect presence of RNF43 protein expression for 63 *BRAF* mutant/MSI, 37 *BRAF* mutant/MSS and 44 *BRAF* wild type cancers from the formalin fixed series. Cytoplasmic staining was least frequent in the *BRAF* mutant/MSI compared to *BRAF* mutant/MSS and *BRAF* wild type cancers (p=0.009) (Table 4A, Figure 3A and 3B). Cytoplasmic RNF43 staining was significantly less common in cancers harbouring the X659fs mutation compared to that in wild type cancers (RNF43 cytoplasmic positive cancers: 11/27, 41% vs 35/49, 71% respectively, p=0.01). No difference in nuclear RNF43 expression was found between the X659fs mutant and wild type cancers (Table 4B).

**Table 4:** Immunohistochemical analysis of *RNF43* expression between cohorts

A. Immunohistochemical analysis of RNF43 cytoplasmic and nuclear expression between cohorts							
	*BRAF* mutant/MSI	*BRAF* mutant/MSS	*BRAF* wild type	p value^A^	p value^B^	p value^C^	p value^D^
Cytoplasm	31/63 (49.2%)	27/37 (73.0%)	33/44 (75.0%)	0.009	0.02	0.009	1.00
Nucleus	36/63 (57.1%)	30/36 (83.3%)	26/43 (60.5%)	0.02	0.008	0.84	<0.05

A p value overall;

B p value between *BRAF* mutant/MSI and *BRAF* mutant/MSS;

C p value between *BRAF* mutant/MSI and *BRAF* wild type;

D p value between *BRAF* mutant/MSS and *BRAF* wild type

**Table:** 

B. Immunohistochemical analysis of RNF43 in cancers harbouring an X659fs mutation							
RNF43 IHC	X659fs mutant n=27	Wild type n=49	P Value
cytoplasmic positive (2-3)	11/27 (40.7%)	35/49 (71.4%)	p=0.01
nuclear positive (2-3)	16/27 (59.3%)	33/27 (67.3%)	p=0.6

β-catenin immunohistochemical staining was assessed, with nuclear accumulation indicative of elevated Wnt signal. Overall, β-catenin was more likely to be nuclear in *BRAF* wildtype compared to *BRAF* mutant cancers (27/28, 96.4% versus 36/92, 39.1%, P<0.0001). In BRAF wild type cancers with nuclear β-catenin staining, 19/27 (70.4%) showed a concomitant increase in RNF43 cytoplasmic expression, which may represent a futile attempt to dampen the Wnt signal by RNF43-mediated degradation of the Frizzled receptor. In *BRAF* mutant/MSI cancers with normal staining for β-catenin, 21/33 (63.6%) also maintained normal RNF43 staining compared to only 4/23 (17.4%) *BRAF* mutant/MSS cancers (P<0.001). This likely reflects the higher *RNF43* mutation rate in MSI cancers and therefore the inability to upregulate *RNF43* in response to elevated Wnt signal, as is common in *BRAF* wild type cancers.

### Response to Porcupine inhibitor, LGK974, for *RNF43* and *ZNRF3* mutant cell lines

**Figure 4:**
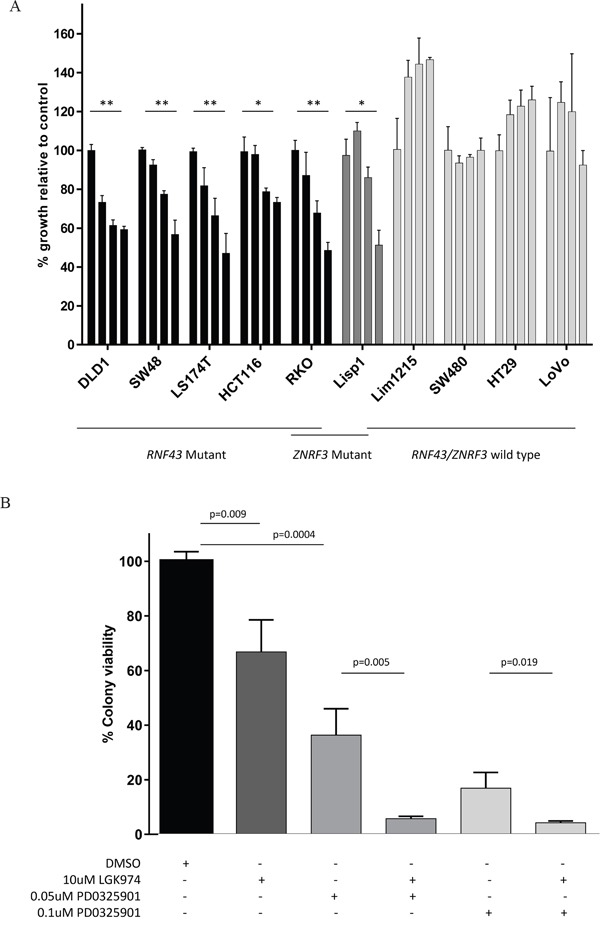
Cell colony growth of colorectal cancer cell lines following treatment with Porcupine inhibitor, LGK974 (A); and in combination with MEK inhibitor, PD0325901 (B) **A.** Cell lines harbouring an *RNF43* and/or *ZNRF3* mutation showed significantly reduced cell colony formation following treatment with the Porcupine inhibitor, LGK974 in a dose dependent manner. There was no decrease of cell colony formation in wild type cell lines with LGK974 treatment. The graph shows the percentage of cell growth relative to the control. The bars per cell line correspond to treatment with 1) control (DMSO), 2)5 uM LGK974, 3)10 uM LGK974 and 4)20 uM LGK974. Significance of decreased cell line growth following LGK974 treatment is shown (** p<0.001; *p<0.01). Standard deviation error bars are shown. **B.** When LGK974 treatment was combined with a MEK inhibitor, PD0325901 in the *RNF43*/*ZNRF3* mutant cell line, RKO, there was further abrogation of cell colony growth in a dose dependent manner compared to either single treatment. Standard deviation error bars are shown.

Cell lines were seeded at varying densities according to growth rate to promote long term colony formation. Treatment with the porcupine inhibitor, LGK974, at various concentrations (at 5uM, 10uM and 20uM) was replenished every 48 hours for approximately 2 weeks and colonies were subsequently fixed, stained and counted by two independent examiners. In all *RNF43* and *ZNRF3* mutant cell lines, colony formation was inhibited in a dose-dependent manner of up to 53% with LGK974 treatment compared to growth seen in the DMSO treated control cells (Figure 4A; Supplementary Figure S1A). HCT116 had the least growth inhibition with LGK974 treatment, whilst RKO and LS174T demonstrated the most. There was no overall decrease of growth with LGK974 treatment observed in any of the *RNF43* / *ZNRF3* wild type cell lines (Figure 4A).

### Combination of Porcupine and MEK inhibitors accentuate growth inhibition in RKO cells

The RKO cell line most closely resembles primary cancers of the serrated pathway as it is *BRAF* mutant, MSI and CIMP high [24, 25]. RKO harbours a homozygous *RNF43* X659 frameshift mutation and two heterozygous *ZNRF3* mutations (X249fs, G481W). Treatment with 10μM LGK974 decreased colony growth by approximately 30% (Figure 4A and 4B), and treatment with the MEK inhibitor, PD0325901, reduced colony growth by 64% at 0.05uM and 84% at 0.1uM (Figure 4B). The combination of LGK974 and PD0325901 synergised to further reduce RKO's cell colony growth (Figure 4B), and this was in line with markedly reduced phosphorylation of ERK1/2 and downstream c-MYC (Supplementary Figure S2). These results demonstrate the potential effectiveness of this novel combination therapy in treating *RNF43* / *BRAF* mutant serrated pathway cancers. (Images of cells treated with 0.002uM – 0.5uM with and without LGK974 application are shown in Supplementary Figure S1B).

## DISCUSSION

The E3 ubiquitin ligases RNF43 and ZNRF3 normally cooperate to downregulate the Wnt signal by targeting the Frizzled receptor for degradation. This study provides a comprehensive analysis of *RNF43* and *ZNRF3* in cohorts of colorectal cancers stratified by *BRAF* mutation and MSI status. The high rate of mutation and structural variation in *BRAF* mutant cancers suggests alteration of these genes is important for progression of serrated pathway cancers, where Wnt activation by *APC* mutation is uncommon [2].

*RNF43* was commonly mutated in *BRAF* mutant cancers compared to *BRAF* wild type cancers. The functional significance of mutations occurring in the proximal region of the gene have previously been established, where Schwank *et al* [26] demonstrated R-spondin independent growth in murine colonic organoids. Here we report 71-80% of MSI cancers from two independent series had an x659 frameshift mutation in the distal region of the gene. This is predicted to result in a premature stop codon and was also reported by Giannakis et al to occur in 64% of MSI cancers [18]. The high mutation rate suggests this mutations confers a positive selective advantage [13]. This was confirmed by Yan et al where biallelic *RNF43* X659fs mutation, along with targeting of *ZNRF3*, in a murine colon organoid model conferred R-spondin independence [27].

Serrated neoplasia may originate in sessile serrated adenomas (SSA) or traditional serrated adenomas (SSA), the former being the precursor of MSI cancers and the latter preceding the development of *BRAF* mutant / MSS cancers [28, 29]. Sekine *et al* [30] reported *RNF43* mutations in 6% SSA and 24% TSA, compared to our findings of 87% in *BRAF* mutant MSI and 24% *BRAF* mutant MSS cancers. This suggests that *RNF43* may assist progression of SSA to MSI cancers, whereas *RNF43* mutation may be involved at an earlier stage of TSA development. *ZNRF3* mutations were not observed in SSAs and in only 1% of TSAs [30] compared to 30% *BRAF* mutant/MSI cancers and 15% *BRAF* mutant /MSS cancers, suggesting that this less common mutation is involved in progression to cancer rather than precursor development. *BRAF* mutant/MSS cancers also commonly showed deletion of the *RNF43* locus at 17q22, which may contribute to gene silencing and compensate for the lower *RNF43* / *ZNRF3* mutation rate in this cancer subgroup.

Cytoplasmic RNF43 expression was least frequently observed in the *BRAF* mutant/MSI cancers. Because RNF43 abrogates the Wnt signal at the level of the Frizzled receptor, it may be expected to be localised predominantly in the cytoplasm or membrane rather than the nucleus. Whether its expression is transient and is degraded soon after functioning, or whether it is maintained within the cell for a period of time is unknown. RNF43 has been reportedly expressed in both the cytoplasm and nucleus of gastric cancer cells [14], the cytoplasm of gastric cancer and glioma cells [31, 32] as well as in the nuclear membrane and endoplasmic reticulum of cervical cancer cells [33]. A recent study described nuclear localization of RNF43 where it functioned to inhibit the Wnt pathway through its sequestering of TCF4, a transcription factor activated by beta-catenin target gene, to the nuclear membrane which prevented TCF4's mediated transcription of genes involved in downstream Wnt signalling [34]. RNF43 may have various time and cell type specific roles each requiring a unique subcellular localization. In the current study, nuclear localisation of β-catenin was used as a surrogate for elevated Wnt signalling. This was observed in the majority of *BRAF* wildtype cancers where elevated cytoplasmic RNF43 staining was also observed, likely as a futile attempt to dampen the Wnt signal by RNF43-mediated degradation of the Frizzled receptor. BRAF wildtype cancers also showed a significant increase in level of transcript expression. MSI cancers were more likely to maintain a normal staining pattern for β-catenin and this was correlated with a lower incidence of abnormal cytoplasmic staining for RNF43, most likely due to the high *RNF43* mutation rate in this subgroup.

The *RNF43* and *ZNRF3* mutant colorectal cancer cell lines were all MSI, and all harbour a mutation within the EGFR-MAPK signalling pathway. Treatment of these cells lines with the Porcupine inhibitor, LGK974, resulted in consistently and significantly reduced cellular proliferation compared to wild type cell lines. The *RNF43* wild type cancer cell lines all harbour either *APC* or β-catenin mutations which would be predicted to render them insensitive to upstream inhibition of the Wnt signal, as was observed. The approximate 50% growth reduction for the *RNF43* and *ZNRF3* mutant cell lines was not as dramatic as was observed for an *RNF43* mutant organoid model, where the colonoid was sensitive to the similar porcupine inhibitor, IWP2 [35]. This difference may reflect a different magnitude of reliance on endogenous Wnt signalling compared to the colorectal cell lines. Organoid models are more representative of *in vivo* cancer growth due to their three dimensional structure and shorter time in culture. The epigenetic instability of *BRAF* mutant cancers in particular can induce DNA methylation and silencing of other Wnt pathway molecules that may alter the sensitivity of the cells to an upstream porcupine inhibitor. The novel combination of Porcupine and MEK inhibition synergistically inhibited RKO cell growth by more than 90%, which suggests this may be a promising therapeutic strategy to treat *BRAF* / *RNF43* / *ZNRF3* mutant serrated pathway cancers.

Overall this study has identified that the E3 ubiquitin ligases, *RNF43* and *ZNRF3*, are frequently mutated in *BRAF* mutant cancers of the serrated pathway, particularly those that are MSI. Heterozygous loss of the *RNF43* and *ZNRF3* loci were identified in *BRAF* mutant/MSS cancers. *BRAF* mutant cancers did not upregulate *RNF43* or *ZNRF3* expression at the transcript or protein level, unlike the *BRAF* wild type cancers that attempt to mitigate the Wnt signal. Functionally, it was shown that *RNF43* as well as *ZNRF3* mutant colorectal cancers may be Wnt ligand dependent and that inhibition of the Wnt signal by targeting Porcupine may facilitate decreased growth of a proportion of MSI serrated pathway cancers. Furthermore, the novel combination of Porcupine and MEK inhibition resulted in considerable growth reduction, which indicates this may be a promising treatment approach for patients with serrated pathway cancers harbouring *RNF43* and/or *ZNRF3* mutations.

## MATERIALS AND METHODS

### Patient demography and sample selection

Cancer and matched normal samples were obtained either as fresh frozen tissue from patients undergoing surgery at the Royal Brisbane and Women's Hospital, or as formalin-fixed paraffin embedded (FFPE) tissue from Envoi Specialist Pathologists, Brisbane, Australia. This study was approved by the Royal Brisbane and Women's Hospital and Bancroft Human Research Ethics Committees.

Clinicopathological data including gender, age at diagnosis and anatomical site of cancer (with proximal defined as proximal to the splenic flexure) were collected. DNA from fresh cancer and matched normal tissue was extracted using AllPrep DNA mini kit (Qiagen, Dusseldorf, Germany). DNA from the FFPE cancers was extracted by the Chelex-100 method (Bio-Rad Laboratories, CA, USA). The presence of MSI had been previously analysed for the RBWH cancer samples using the National Cancer Institute's 5 marker panel [21, 36, 37]. Cancers from Envoi Specialist Pathologists were evaluated for immunohistochemical loss of MLH1 mismatch repair protein expression as a surrogate for MSI. Presence of the *BRAF* V600E (a1796t) mutation, *p53* mutation (over exons 4-8) and *KRAS* mutation (over codons 2 and 3) had been previously investigated for the fresh RBWH samples [21] whilst *BRAF* V600E (a1796t) and *KRAS* (codons 2 and 3) mutations were analysed for the formalin fixed Envoi samples as previously described [21, 38–40].

Cell LinesA panel of colorectal cancer cell lines: DLD1, HCT116, HT29, Lim1215, LISP1, LoVo, LS174T, RKO, SW48 and SW480 were maintained in RPMI media supplemented with 10% fetal bovine serum (Bovogen Biologicals, Victoria, Australia) and 1% penicillin-streptomycin (Life Technologies, Carlsbad, USA). Cell lines were authenticated by short tandem repeat profiling to confirm authenticity of ATCC cell lines.

### *RNF43* and *ZNRF3* mutation analysis

Sanger sequencing was analysed across the coding region of *RNF43* (performed by Macrogen Incorporated, South Korea) in 87 *BRAF* mutant (54 MSI and 33 MSS) and 79 *BRAF* wild type cancers from the RBWH series. *ZNRF3* was sequenced in all 87 *BRAF* mutant and an unselected subset of 27 *BRAF* wild type cancers. The second series of FFPE cancers from Envoi Specialist Pathologists consisting of 48 *BRAF* mutant (36 MSI and 12 MSS) and 29 *BRAF* wild type cancers were sequenced to further validate the frequency of the X659fs mutation (sequencing primers: F 5′GTCCAGGCCTCCTATTCCTC; R 5′ CTGGTAGCAGCCTCTTGTCC). Only mutations predicted to elicit a functional response (frameshift, missense, nonsense and splice mutations) were included in analyses, and mutations found intronically, in untranslated regions or silent mutations were excluded from further investigation.

### Transcript expression

19 *BRAF* mutant (13 MSI, 6 MSS), 27 *BRAF* wild type and 12 matched normal mucosa samples were randomly chosen from the freshly collected RBWH series and analysed for transcript expression on the HumanHT-12 v4 Expression BeadChip arrays (Illumina, San Diego, CA). Total mRNA (500 ng) was reverse-transcribed, amplified and biotinylated using the Illumina TotalPrep-96 RNA Amp Kit (Ambion, Austin, TX). The labelled cRNA (750 ng) was hybridized to the BeadChip arrays followed by washing, blocking, and staining with streptavidin-Cy3 according to the manufacturer's specifications. Fluorescence intensity for each probe on the array chips were measured on the iScan system and the data was extracted using GenomeStudio software (Illumina). All arrays passed quality control criteria in the GenomeStudio package as described by the manufacturer. A sample probe profile file generated in GenomeStudio without background subtraction or normalisation was used for subsequent analysis in R. The data was background corrected and quantile normalised using the limma package function. Probes with detection p-values P>0.05 in >95% of all samples were excluded from further analysis.

### Single nucleotide polymorphism arrays

33 *BRAF* mutant/MSS, 30 *BRAF* mutant/MSI, 18 *BRAF* wild type/MSS cancers and matched normal samples all randomly chosen from the RBWH series were analysed for genome-wide copy number aberrations (CNAs) with HumanCytoSNP-12v2.1 Single Nucleotide Polymorphism (SNP) arrays (Illumina, San Diego, Ca.) according to the manufacturer's instructions. The beadchips were scanned using Illumina's iScan system and the image data was analysed with Illumina's GenomeStudio version 2011.1.0.24550 and as previously described [22].

### Immunohistochemical analysis

Tissue sections obtained from FFPE blocks from the Envoi series underwent antigen retrieval at low pH (pH6, Reveal decloaker; Biocare Medical, CA, USA) for 15mins at 105°C. H_2_O_2_ and Sniper were used to facilitate endogenous peroxidase and protein blocks respectively. Primary antibodies were manually applied and incubated: RNF43 antibody (anti-RNF43 HPA-008079; Sigma, St Louis, MO, USA) at 1/500 dilution for 1 hour and beta-catenin (anti-beta-catenin 224M16 [14] Cell Marque, California, USA) at 1/600 for 1 hour. MACH3 rabbit or mouse secondary antibody probe and polymer were applied for 15 and 30 minutes respectively (Biocare Medical, CA, USA), and DAB chromagen (Biocare Medical, CA, USA) was applied for 5-8 minutes.

Observation of samples was performed by an expert gastrointestinal pathologist (MB) and scored on a scale of 0-3 with 0 representing absent and 3 representing maximal staining. A score of 0-1 was considered negative and a score of 2-3 as positive. RNF43 staining within the cancer region of a sample section was compared to that observed within normal lymphocytes, which served as an internal control.

### Cell colony assays

All cell lines were authenticated using short tandem repeat (STR) profiling in accordance with ATCC standards. Cell lines were last tested in June 2015.

Cell lines were seeded at varying densities in 6 well plates (HCT116, SW480 at 500 cells/well; DLD1, RKO, Lim1215 at 750 cells/well; SW48, Lisp1, LS174T, LoVo, HT29 at 1000 cells/well) and maintained in 2mls RPMI media as described above. 24 hours later, cells were treated in triplicate with either DMSO in control wells or the Porcuine inhibitor LGK974 (at 5uM, 10uM or 20uM in DMSO) (Cayman Chemicals, Michigan, USA) every 48 hours. At approximately 2 weeks, cells were fixed with ice-cold 100% methanol and stained with 0.5% crystal violet in 80% methanol. For LGK974 treatments, cell colonies were counted by 2 examiners and the counts were averaged. For combination treatments, RKO cells were treated at 24 hours post plating and then every 48 hours with either DMSO, 10uM LGK974, and/or the MEK inhibitor PD0325901 (Selleck Chemicals, Texus, USA) (at 0.002uM, 0.05uM, 0.1uM, 0.3uM or 0.5uM). Drug combination cell colony assays were quantitated using Aperio ImageScope v12.1.0.5019 with application of the Positive Pixel Count v9.1 algorithm.

Western blotting1X10^6^ RKO cells were either treated singly or in-combination with LGK974 and PD0325901. DMSO used as a vehicle control. 48 hr post-treatment, cells were collected and lysed with 7M urea buffer (7M Urea, 1% SDS, 20-30 mM Tris pH 8, 100-150 mM NaCl). Western blotting was then performed according to standard protocol as described previously [41] using indicated antibodies. C-MYC (AB32072, Abcam), pERK1/2 (#4370), ERK1/2 (#4695), β-catenin (#9582, cell signalling) and Cox-IV (PN926-42214, Li-COR). The Super Signal chemiluminescent ECL-plus (Amersham) was used.

### Statistical analysis

Categorical data was analysed for significance using Fisher's exact test or Pearson's chi-squared test where appropriate. Continuous data was analysed with either a student's t-test or ANOVA as appropriate. P values <0.05 were considered significant.

## SUPPLEMENTARY FIGURES AND TABLES




