# Supplementary material for: RNF43 and ZNRF3 are commonly altered in serrated pathway colorectal tumorigenesis
Source: Oncotarget. 2016 Sep 20;7(43):70589–600. doi: 10.18632/oncotarget.12130 (PMC5342576; doi:10.18632/oncotarget.12130)
Supplement: Supplementary file 1 [file oncotarget-07-70589-s001.pdf]

RNF43 and *ZNRF3* are commonly altered in serrated pathway colorectal tumorigenesis

SUPPLEMENTARY FIGURES AND TABLES

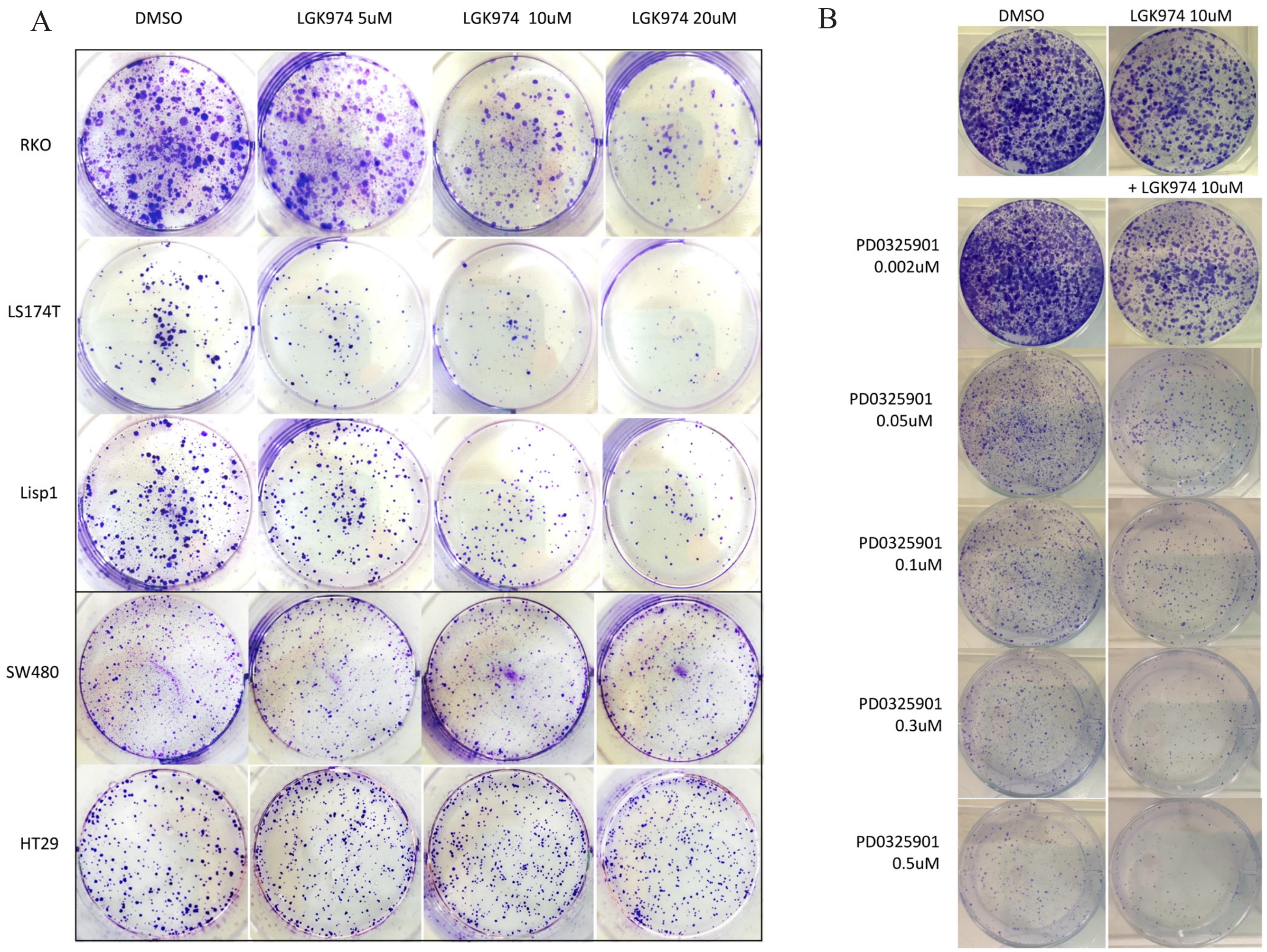

Supplementary Figure S1: A. Examples of cell colony images showing increasing inhibition of growth of *RNF43*/*ZNRF3* mutant cell lines (RKO, LS174T, Lisp1) following treatment with increasing concentrations of Porcupine inhibitor (LGK974), compared to unaffected growth of wild type cell lines (SW480, HT29). **B.** Cell colony images showing treatment of RKO with MEK inhibitor (PD0325901) 0.002uM – 0.5uM with and without application of 10uM LGK974. From 0.05uM PD0325901 treatment there was inhibition of growth. This was further accentuated with combination of 10uM LGK974 treatment.

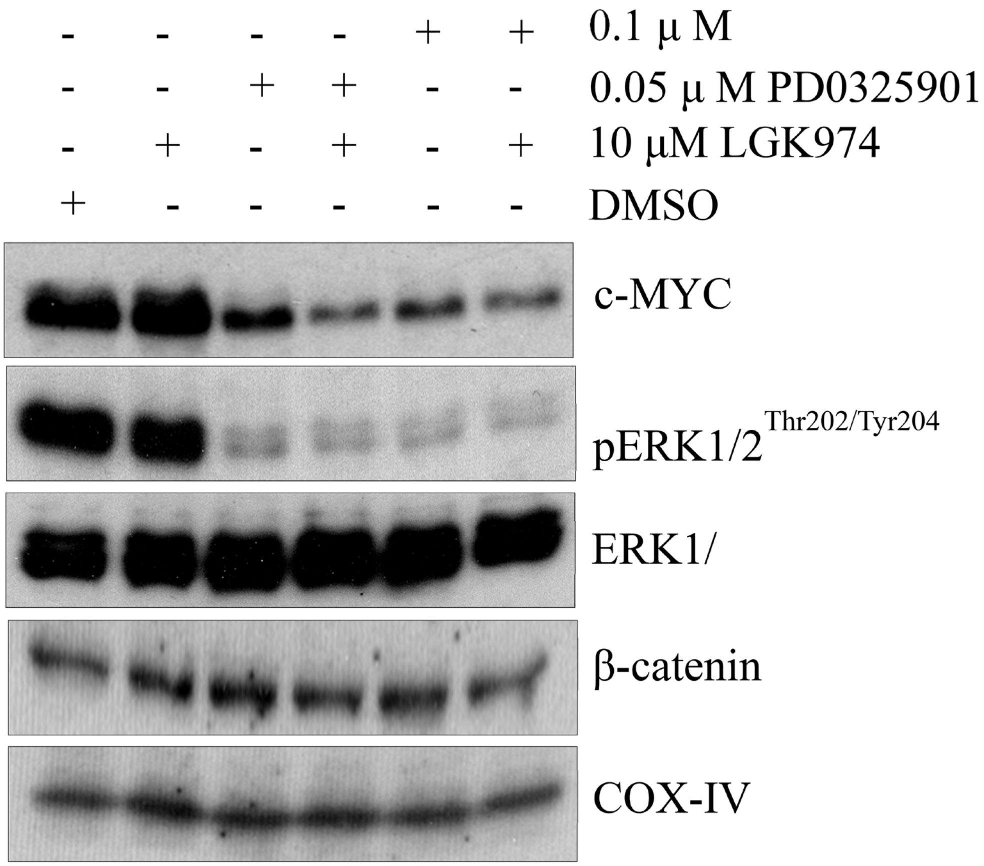

Supplementary Figure S2: RKO cells were plated and treated with either DMSO, the Porcupine inhibitor LGK974 (at 10uM), and/or the MEK inhibitor PD0325901 (at 0.1uM or 0.05uM). At 48 hour post-treatment, cells were lysed and protein was extracted. Western blots show that upon MEK1/2 and PD0325901 inhibition, there was a marked decrease of phospho- ERK1/2 and c-MYC levels. COX-IV was used as a loading control.

Supplementary Table S1: Clinical and molecular data according to A) *RNF43* and B) *ZNRF3* mutation status per cohort

See Supplementary File 1

Supplementary Table S2: A) *RNF43* transcript expression values of cancers with *RNF43* mutation data. (*BRAF* mutant/MSI n=13, *BRAF* mutant/MSS n=6, *BRAF* wild type n=27)

B) *ZNRF3* transcript expression values of cancers with *ZNRF3* mutation data. (*BRAF* mutant/MSI n=13, *BRAF* mutant/MSS n=6, *BRAF* wild type n=27)

See Supplementary File 1
