# Supplementary material for: RNF43 and ZNRF3 are commonly altered in serrated pathway colorectal tumorigenesis
Source: Oncotarget. 2016 Sep 20;7(43):70589–600. doi: 10.18632/oncotarget.12130 (PMC5342576; doi:10.18632/oncotarget.12130)
Supplement: Supplementary file 2 [file oncotarget-07-70589-s002.docx]

**Supplementary Data - *RNF43* and *ZNRF3* are Commonly Altered in Serrated Pathway Colorectal Tumorigenesis**

Table 1 – Clinical and molecular data according to A) *RNF43* and B) *ZNRF3* mutation status per cohort

| A. *RNF43* |  |  |  |  |  |  |  |  |  |
| --- | --- | --- | --- | --- | --- | --- | --- | --- | --- |
|  | *BRAF*mut/MSI. *RNF43* mutant | *BRAF*mut/MSI. *RNF43* wt | p value | *BRAF*mut/MSS. *RNF43* mutant | *BRAF*mut/MSS. *RNF43* wt | p value | *BRAF*wt/MSS. *RNF43* mutant | *BRAF*wt/MSS. *RNF43* wt | p value |
| n | 46 (85.2%) | 8 (14.8%) | - | 8 (24.3%) | 25 (75.8%) | - | 3 (3.8%) | 76 (96.2%) | - |
| Average age (yrs) | 75.7 | 68.4 | 0.09 | 68.1 | 68.5 | 0.75 | 58.5 | 68.6 | 0.15 |
| Female gender | 42/46 (81.3%) | 5/8 (62.5%) | 0.06 | 3/8 (37.5%) | 14/25 (56.0%) | 0.44 | 0/3 (0%) | 30/76 (39.5%) | 0.28 |
| Proximal site | 20/21 (95.2%) | 5 /6 (83.3%) | 0.40 | 6/8 (75.0%) | 11/16 (68.8%) | 1.00 | 2/2 (100%) | 15/61 (24.6%) | 0.07 |
| Stage (AJCC I/II) | 28/40 (70.0%) | 4/6 (66.7%) | 1.00 | 2/8 (25.0%) | 5/17 (29.4%) | 1.00 | 0/3 (0%) | 35/69 (50.7%) | 0.24 |
| CIMP high | 46/46 (100%) | 8/8 (100%) | 1.00 | 7/8 (87.5%) | 25/25 (100%) | 0.24 | 0/3 (0%) | 12/76 (15.8%) | 1.00 |
| *p53* mutant | 9/39 (23.1%) | 1/7 (14.2%) | 1.00 | 3/8 (37.5%) | 9/23 (39.1%) | 1.00 | 1/3 (33.3%) | 26/65 (40.0%) | 1.00 |
| *PIK3CA* mutant | 6/37 (16.2%) | 1/8 (12.5%) | 1.00 | 1/8 (12.5%) | 4/23 (17.4%) | 1.00 | 0/3 (0%) | 12/72 (16.7%) | 1.00 |
| *MGMT* methylated | 20/40 (50.0%) | 3/5 (60.0%) | 1.00 | 2/8 (25.0%) | 4/24 (16.7%) | 0.62 | 1/3 (33.3%) | 20/69 (29.0%) | 1.00 |
| *KRAS* mutant | 0/46 (0%) | 0/8 (0%) | 1.00 | 0/8 (0%) | 0/25 (0%) | 1.00 | 1/3 (33.3%) | 41/76 (53.9%) | 0.60 |
| *ZNRF3* mutant | 15/46 (32.6%) | 1/8 (12.5%) | 0.41 | 3/8 (37.5%) | 2/25 (8.0%) | 0.08 | 0/3 (0%) | 0/76 (0%) | 1.00 |
|  |  |  |  |  |  |  |  |  |  |
|  |  |  |  |  |  |  |  |  |  |
| B. *ZNRF3*: |  |  |  |  |  |  |  |  |  |
|  | *BRAF*mut/MSI. *ZNRF3* mutant | *BRAF*mut/MSI. *ZNRF3* wt | p value | *BRAF*mut/MSS. *ZNRF3* mutant | *BRAF*mut/MSS. *ZNRF3* wt | p value | *BRAF*wt/MSS. *ZNRF3* mutant | *BRAF*wt/MSS. *ZNRF3* wt | p value |
| n | 16/54 (29.6%) | 38/54 (71.4%) | - | 5/33 (15.2%) | 28/33 (84.8%) | - | 0/27 (0%) | 27/27 (100%) | - |
| Average age (yrs) | 77.7 | 73.3 | 0.20 | 70.1 | 68.1 | 0.68 | - | 68.2 | - |
| Female gender | 13/16 (81.3%) | 29/38 (76.3%) | 1.00 | 0/5 (0%) | 17/28 (60.7%) | **0.02** | - | 13/27 (48.1%) | - |
| Proximal site | 11/12 (91.7%) | 31/33 (93.9%) | 1.00 | 1/4 (25.0%) | 16/19 (84.2%) | **0.04** | - | 5/25 (20.0%) | - |
| Stage (AJCC I/II) | 6/9 (66.7%) | 25/34 (73.5%) | 0.69 | 1/5 (20.0%) | 6/20 (30.0%) | 1.00 | - | 9/27 (33.3%) | - |
| CIMP high | 16/16 (100%) | 38/38 (100%) | 1.00 | 5/5 (100%) | 27/28 (96.4%) | 1.00 | - | 0/27 (0%) | - |
| *p53* mutant | 1/11 (9.1%) | 9/34 (26.5%) | 0.41 | 2/5 (40.0%) | 10/26 (38.5%) | 1.00 | - | 10/27 (37.0%) | - |
| *PIK3CA* mutant | 2/11 (18.2%) | 5/33 (15.2%) | 1.00 | 1/5 (10.0%) | 4/26 (15.4%) | 1.00 | - | 4/27 (14.8%) | - |
| *MGMT* methylated | 6/12 (50.0%) | 17/33 (51.5%) | 1.00 | 2/5 (40.0%) | 4/27 (14.8%) | 0.23 | - | 5/27 (18.5%) | - |
| *KRAS* mutant | 0/16 (0%) | 0/38 (0%) | 1.00 | 0/5 (0%) | 0/28 (0%) | 1.00 | - | 17/27 (63.0%) | - |
| *RNF43* mutant | 15/16 (93.8%) | 31/38 (81.6%) | 0.41 | 3/5 (60.0%) | 5/28 (17.9%) | 0.08 | - | 3/27 (11.1%) | - |
|  |  |  |  |  |  |  |  |  |  |
|  |  |  |  |  |  |  |  |  |  |

Table 2

A) *RNF43* transcript expression values of cancers with *RNF43* mutation data. (*BRAF* mutant/MSI n=13, *BRAF* mutant/MSS n=6, *BRAF* wild type n=27)

| **Cohort** | **Sample** | **RNF43 Mutant (1=mutant, 0=wild type)** | **Type of RNF43 mutation (2=X659fs, 1=other mutation, 0=wild type)** | **RNF43 Expression value** |
| --- | --- | --- | --- | --- |
| BRAF mutant/MSI | CG160 | 1 | 2 | 8.01 |
| BRAF mutant/MSI | CG268 | 1 | 2 | 6.71 |
| BRAF mutant/MSI | CG284 | 0 | 0 | 7.57 |
| BRAF mutant/MSI | CG320 | 1 | 2 | 6.09 |
| BRAF mutant/MSI | CG337 | 0 | 0 | 7.07 |
| BRAF mutant/MSI | CG341 | 1 | 2 | 6.53 |
| BRAF mutant/MSI | CG378 | 0 | 0 | 7.96 |
| BRAF mutant/MSI | CG389 | 1 | 2 | 6.74 |
| BRAF mutant/MSI | CG426 | 1 | 2 | 6.16 |
| BRAF mutant/MSI | CG432 | 1 | 2 | 5.16 |
| BRAF mutant/MSI | G1336 | 1 | 2 | 6.46 |
| BRAF mutant/MSI | G1679 | 0 | 0 | 5.82 |
| BRAF mutant/MSI | G1802 | 1 | 2 | 6.63 |
| BRAF mutant/MSS | CG212 | 0 | 0 | 8.22 |
| BRAF mutant/MSS | CG304 | 0 | 0 | 8.89 |
| BRAF mutant/MSS | G1347 | 1 | 1 | 8.33 |
| BRAF mutant/MSS | G1383 | 0 | 0 | 7.89 |
| BRAF mutant/MSS | G1623 | 0 | 0 | 4.65 |
| BRAF mutant/MSS | G1627 | 1 | 1 | 7.06 |
| BRAF wild type | CG075 | 0 | 0 | 9.05 |
| BRAF wild type | CG078 | 0 | 0 | 9.14 |
| BRAF wild type | CG080 | 0 | 0 | 7.82 |
| BRAF wild type | CG081 | 0 | 0 | 9.78 |
| BRAF wild type | CG096 | 0 | 0 | 9.20 |
| BRAF wild type | CG104 | 0 | 0 | 8.93 |
| BRAF wild type | CG119 | 0 | 0 | 7.99 |
| BRAF wild type | CG121 | 0 | 0 | 9.28 |
| BRAF wild type | CG123 | 0 | 0 | 9.14 |
| BRAF wild type | CG127 | 0 | 0 | 9.16 |
| BRAF wild type | CG128 | 0 | 0 | 8.51 |
| BRAF wild type | CG131 | 0 | 0 | 8.70 |
| BRAF wild type | CG132 | 0 | 0 | 8.93 |
| BRAF wild type | CG138 | 0 | 0 | 8.91 |
| BRAF wild type | CG145 | 0 | 0 | 10.04 |
| BRAF wild type | CG149 | 0 | 0 | 8.94 |
| BRAF wild type | CG174 | 1 | 1 | 9.06 |
| BRAF wild type | CG189 | 0 | 0 | 9.58 |
| BRAF wild type | CG190 | 0 | 0 | 9.66 |
| BRAF wild type | CG208 | 0 | 0 | 9.64 |
| BRAF wild type | CG214 | 0 | 0 | 6.65 |
| BRAF wild type | G1282 | 0 | 0 | 8.58 |
| BRAF wild type | G1329 | 0 | 0 | 9.01 |
| BRAF wild type | G1332 | 0 | 0 | 7.50 |
| BRAF wild type | G1337 | 0 | 0 | 7.50 |
| BRAF wild type | G1755 | 0 | 0 | 9.49 |
| BRAF wild type | G1778 | 0 | 0 | 9.31 |

B) *ZNRF3* transcript expression values of cancers with *ZNRF3* mutation data. (*BRAF* mutant/MSI n=13, *BRAF* mutant/MSS n=6, *BRAF* wild type n=27)

| **Cohort** | **Sample** | **ZNRF3 Mutant (1=mutant, 0=wild type)** | **ZNRF3 Expression value** |
| --- | --- | --- | --- |
| BRAF mutant/MSI | CG160 | 1 | 5.68 |
| BRAF mutant/MSI | CG268 | 0 | 6.06 |
| BRAF mutant/MSI | CG284 | 0 | 7.14 |
| BRAF mutant/MSI | CG320 | 0 | 5.97 |
| BRAF mutant/MSI | CG337 | 1 | 6.67 |
| BRAF mutant/MSI | CG341 | 1 | 6.44 |
| BRAF mutant/MSI | CG378 | 0 | 6.68 |
| BRAF mutant/MSI | CG389 | 1 | 5.64 |
| BRAF mutant/MSI | CG426 | 1 | 5.97 |
| BRAF mutant/MSI | CG432 | 0 | 5.60 |
| BRAF mutant/MSI | G1336 | 0 | 6.05 |
| BRAF mutant/MSI | G1679 | 0 | 6.63 |
| BRAF mutant/MSI | G1802 | 0 | 6.66 |
| BRAF mutant/MSS | CG212 | 0 | 8.10 |
| BRAF mutant/MSS | CG304 | 0 | 7.19 |
| BRAF mutant/MSS | G1347 | 0 | 5.84 |
| BRAF mutant/MSS | G1383 | 1 | 5.85 |
| BRAF mutant/MSS | G1623 | 0 | 6.73 |
| BRAF mutant/MSS | G1627 | 1 | 6.89 |
| BRAF wild type | CG075 | 0 | 6.99 |
| BRAF wild type | CG078 | 0 | 7.04 |
| BRAF wild type | CG080 | 0 | 6.69 |
| BRAF wild type | CG081 | 0 | 7.92 |
| BRAF wild type | CG096 | 0 | 7.52 |
| BRAF wild type | CG104 | 0 | 7.22 |
| BRAF wild type | CG119 | 0 | 6.87 |
| BRAF wild type | CG121 | 0 | 7.68 |
| BRAF wild type | CG123 | 0 | 7.81 |
| BRAF wild type | CG127 | 0 | 7.36 |
| BRAF wild type | CG128 | 0 | 6.00 |
| BRAF wild type | CG131 | 0 | 6.72 |
| BRAF wild type | CG132 | 0 | 7.80 |
| BRAF wild type | CG138 | 0 | 7.18 |
| BRAF wild type | CG145 | 0 | 8.83 |
| BRAF wild type | CG149 | 0 | 8.24 |
| BRAF wild type | CG174 | 0 | 7.98 |
| BRAF wild type | CG189 | 0 | 7.10 |
| BRAF wild type | CG190 | 0 | 7.60 |
| BRAF wild type | CG208 | 0 | 7.22 |
| BRAF wild type | CG214 | 0 | 6.20 |
| BRAF wild type | G1282 | 0 | 7.06 |
| BRAF wild type | G1329 | 0 | 5.46 |
| BRAF wild type | G1332 | 0 | 6.41 |
| BRAF wild type | G1337 | 0 | 6.65 |
| BRAF wild type | G1755 | 0 | 7.02 |
| BRAF wild type | G1778 | 0 | 8.45 |
